# A Three-Day Prehabilitation Program is Cost-Effective for Preventing Pulmonary Complications after Heart Valve Surgery: A Health Economic Analysis of a Randomized Trial

**DOI:** 10.31083/j.rcm2509323

**Published:** 2024-09-10

**Authors:** Wei Huang, Yuqiang Wang, Zeruxin Luo, Xiu Zhang, Mengxuan Yang, Jianhua Su, Yingqiang Guo, Pengming Yu

**Affiliations:** ^1^Department of Rehabilitation Medicine, West China Hospital, Sichuan University, 610041 Chengdu, Sichuan, China; ^2^Department of Cardiac Surgery, West China Hospital, Sichuan University, 610041 Chengdu, Sichuan, China

**Keywords:** cost-effectiveness analysis, postoperative pulmonary complications, valve heart disease, prehabilitation

## Abstract

**Background:**

While prehabilitation (pre surgical exercise) effectively prevents postoperative pulmonary complications (PPCs), its cost-effectiveness in valve heart disease (VHD) remains unexplored. This study aims to evaluate the cost-effectiveness of a three-day prehabilitation program for reducing PPCs and improving quality adjusted life years (QALYs) in Chinese VHD patients.

**Methods:**

A cost-effectiveness analysis was conducted alongside a randomized controlled trial featuring concealed allocation, blinded evaluators, and an intention-to-treat analysis. In total, 165 patients scheduled for elective heart valve surgery at West China Hospital were randomized into intervention and control groups. The intervention group participated in a three-day prehabilitation exercise program supervised by a physiotherapist while the control group received only standard preoperative education. Postoperative hospital costs were audited through the Hospital Information System, and the EuroQol five-dimensional questionnaire was used to provide a 12-month estimation of QALY. Cost and effect differences were calculated through the bootstrapping method, with results presented in cost-effectiveness planes, alongside the associated cost-effectiveness acceptability curve (CEAC). All costs were denominated in Chinese Yuan (CNY) at an average exchange rate of 6.73 CNY per US dollar in 2022.

**Results:**

There were no statistically significant differences in postoperative hospital costs (8484 versus 9615 CNY, 95% CI –2403 to 140) or in the estimated QALYs (0.909 versus 0.898, 95% CI –0.013 to 0.034) between the intervention and control groups. However, costs for antibiotics (339 versus 667 CNY, 95% CI –605 to –51), nursing (1021 versus 1200 CNY, 95% CI –330 to –28), and electrocardiograph monitoring (685 versus 929 CNY, 95% CI –421 to –67) were significantly lower in the intervention group than in the control group. The CEAC indicated that the prehabilitation program has a 92.6% and 93% probability of being cost-effective in preventing PPCs and improving QALYs without incurring additional costs.

**Conclusions:**

While the three-day prehabilitation program did not significantly improve health-related quality of life, it led to a reduction in postoperative hospital resource utilization. Furthermore, it showed a high probability of being cost-effective in both preventing PPCs and improving QALYs in Chinese patients undergoing valve surgery.

**Clinical Registration Number:**

This trial is registered in the Chinese Clinical Trial Registry (URL: https://www.chictr.org.cn/) with the registration identifier ChiCTR2000039671.

## 1. Introduction

Valve heart disease (VHD) continues to be a significant healthcare challenge in China, affecting an estimated 25 million patients [1]. The burden of this condition is expected to grow along with the demographic shift towards an older population [1, 2]. While surgery is the preferred treatment for patients with VHD, it carries significant postoperative risks [3, 4, 5, 6]. These postoperative pulmonary complications (PPCs), with incidences ranging from 10% to 72%, significantly affect patient outcomes [7, 8, 9, 10, 11]. These complications have far-reaching effects, including extended postoperative intensive care unit (ICU) stays, higher readmission rates [7, 12, 13, 14], and consequently increased healthcare costs and resource demands [14, 15].

Prehabilitation is designed to improve a patient’s functional status before surgery, aiming to reduce morbidity and facilitate recovery [16, 17, 18], encompassing respiratory therapy, exercise, and multidisciplinary interventions [19]. The adoption of prehabilitation has surged over the last decade, bolstered by substantial evidence for its efficacy and safety [18, 19, 20]. Furthermore, it has been integrated into the Enhanced Recovery After Surgery (ERAS) protocol, underscoring its significance in improving surgical outcomes [21].

Despite the clinical advantages of prehabilitation, its economic impact, particularly for heart valve surgery patients, remains largely unexplored. Economic studies have largely focused on abdominal, oncological surgeries, or cancer patients [22, 23, 24], with little attention to address the cost-effectiveness of prehabilitation in heart valve surgery. Our prior research demonstrated the feasibility and efficacy of a three-day preoperative multimodal program for Chinese VHD patients undergoing heart valve surgery [25]. Consequently, this study evaluates the cost-effectiveness of this three-day preoperative rehabilitation program in reducing PPCs and enhancing quality-adjusted life years (QALYs) for Chinese VHD patients undergoing valve surgery, in comparison to standard care, from a hospital’s perspective.

## 2. Randomized Controlled Trial (RCT) Methodology

**Fig. 1.**
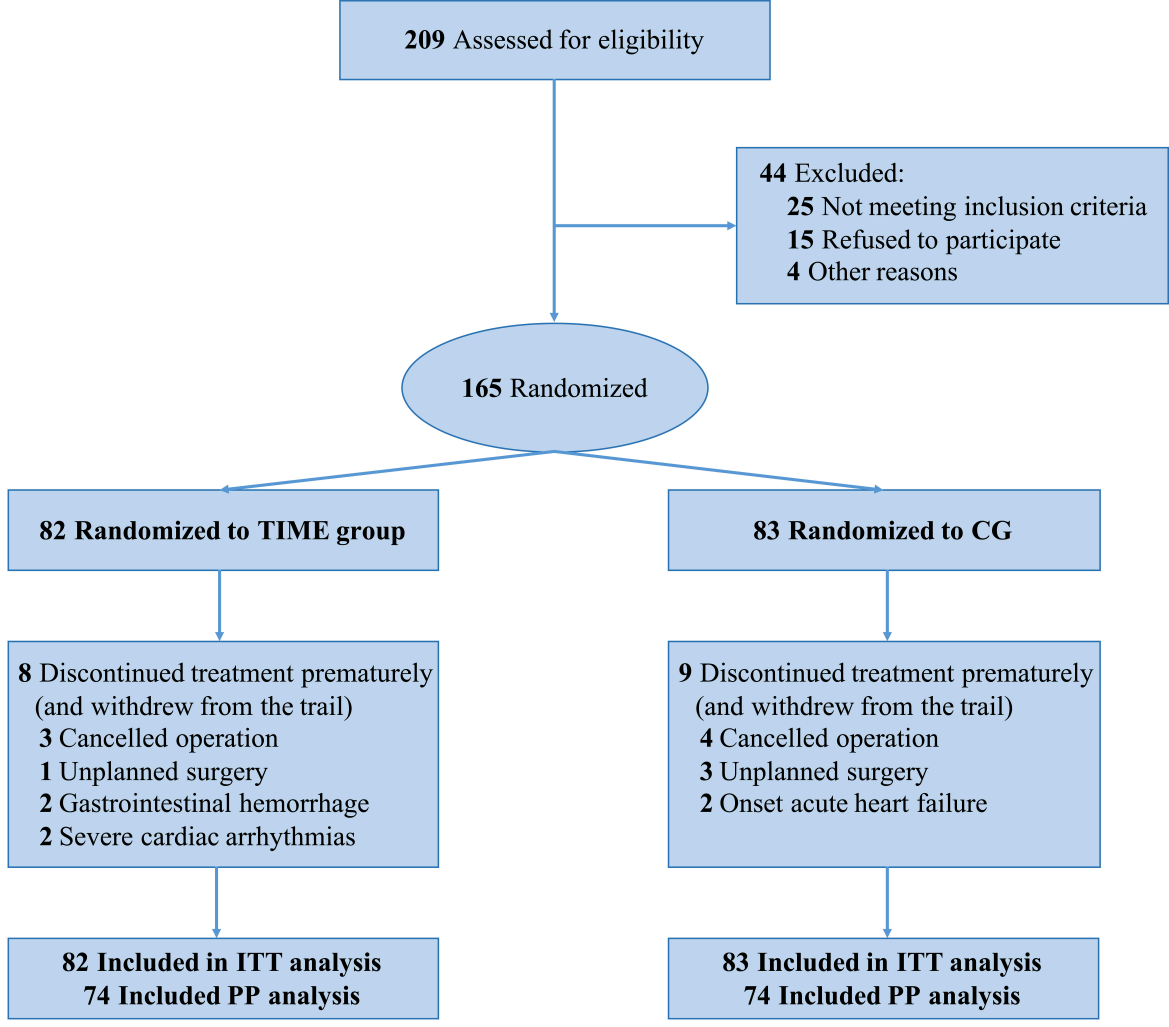
**Patient allocation in the prehabilitation efficacy study**. This figure illustrates the flow of participants through the stages of the study assessing the efficacy of a preoperative multimodal prehabilitation program (three-day of inspiratory muscle training, aerobic muscle training, and specific education (TIME) group) versus standard care (CG group) in patients undergoing valve heart surgery. Initially, 209 individuals were assessed for eligibility, with 44 excluded due to various criteria, resulting in 165 patients randomized into two groups: 82 in the TIME group and 83 in the CG (control group). Both groups had participants who discontinued or withdrew (8 in the TIME group and 9 in the CG group), leading to the final analysis. The intention-to-treat (ITT) analysis included all randomized patients (82 in TIME and 83 in CG), while the per-protocol (PP) analysis accounted for 74 patients in each group who completed the study as per protocol requirements.

This economic evaluation was carried out alongside a RCT and reported in accordance with the Consolidated Health Economic Evaluation Reporting Standards (CHEERS) [26]. The trial flow chart is shown in Fig. 1. Detailed information on the design and methodology of the RCT is available in our previous publication [25]. Briefly, the RCT assessed the efficacy of the prehabilitation program in preventing PPCs among 165 patients undergoing heart valve surgery at the West China Hospital, Sichuan University, China. Participants were randomized into a control group (n = 83) and intervention group (n = 82). Doctors, nurses, assessors and auditors were blinded to group assignments.

### 2.1 Participants

Eligible patients met the following inclusion criteria: an age between 18–90 years, New York Heart Association (NYHA) functional class II to III, and were scheduled for elective heart valve surgery. Exclusion criteria ruled out individuals with cardiovascular instability, pre-surgical pulmonary infection or severe atelectasis, infective endocarditis, aortic aneurysm, aortic dissection, or previous prehabilitation interventions within the preceding 8 weeks.

### 2.2 Interventions

Both groups received the usual preoperative education regarding the surgical procedure, pain management, and the use of drains and catheters, and were delivered by a doctor and a cardiac nurse. An informative booklet was also provided to participants.

Patients in the intervention group were administered an additional three-day prehabilitation program, comprising inspiratory muscle training, aerobic muscle training, and specific education (TIME). The TIME program included 12 sessions, totaling 240 minutes.

The inspiratory muscle training (IMT) component involved six 20-minute sessions (twice daily) using a resistance IMT device (Digi IMT X1, XEEX Co., Ltd., Xiamen, China) set at 30% of maximal inspiratory pressure. A 20-minute specific education session, supervised by a physiotherapist, was provided daily following one of the IMT sessions, either in the morning or afternoon, for three days. The specific education was designed to ensure that patients fully understood key concepts, including the prevention of PPCs, deep breathing and coughing techniques, and self-stretching exercises. It also highlighted the importance of preoperative physical activity, reduced daytime bed rest, early mobilization, and self-directed breathing exercises for ICU and cardiac ward stays.

Aerobic muscle training consisted of daily 20-minute walking sessions in the hospital corridor, with intensity adjusted to 60% of heart rate reserve based on the 6-minute walk test at admission.

### 2.3 Implementation

The TIME program was implemented by a multidisciplinary team including doctors, cardiac nurses, and physiotherapists. The physiotherapist was the primary program lead, while the cardiac nurse facilitated coordination and communication. A doctor supervised patient safety throughout the program.

### 2.4 Outcome Measures

Patients underwent follow-up within the first 14 days following their surgery, during which the length of ICU stay, any rehospitalizations, and postoperative complications were carefully recorded. The evaluation of PPC incidence was conducted in accordance with the criteria from the Kroenke *et al*. study [27], where PPCs were diagnosed based on symptoms, classified into four levels. A diagnosis of PPCs was made when patients exhibited either two or more symptoms at grade 2 or at least one symptom at grade 3 or 4 (**Supplementary File 1**).

Representing the duration of time a patient is in a specific health state, QALYs were estimated by quantifying the area under the curve. A score of zero indicates death, while a score of one represents perfect health [28]. A model was used to project the patient’s QALYs one year after surgery, assuming a two-stage postoperative health trajectory [24]. The first stage, covering the initial 4 weeks post-surgery, was characterized by surgical shock. The second stage, from postoperative week 4 to 52 [29], involved a gradual return to the pre-operative health state. The health state at week 52 was estimated based on baseline levels at hospital admission. The 12-month postoperative QALYs were calculated as the sum of these two stages. Health utility values were derived from the EuroQol five-dimensional questionnaire (EQ-5D) [30], a validated tool for assessing health-related quality of life (HRQoL) in valve surgery patients [31].

### 2.5 Cost Parameters

All costs were presented in Chinese Yuan (CNY) for the year 2022 (the average exchange rate: 1 US dollar = 6.73 CNY). Since we only audited the costs during the patient’s hospital stay, the discount rate was not utilized.

In the context of China’s healthcare system, auditing the specific costs of each element of the preoperative TIME program presented numerous challenges. For instance, materials for booklets were not directly accounted for, and calculating salaries based on working hours for nurses, doctors, or physiotherapists was complex. Additionally, patients were not required to purchase expensive equipment like IMT devices or monitoring equipment for aerobic training. Instead, these were provided by the hospital and were not included in the cost calculation. The cost of the TIME program was based on session rates: 44 CNY per IMT session, 60 CNY per aerobic muscle training session, and 44 CNY per education session. With 12 sessions in total (6 IMT, 3 aerobic training, and 3 education), the total charge for the TIME program was 600 CNY per participant. Usual care costs were not audited in either group as they followed routine medical pathways.

For postoperative hospital costs, we referenced a study by Boden *et al*. [24] conducted in Australia and New Zealand. These costs included ICU and cardiac ward stays, respiratory support, chest imaging, electrocardiogram (ECG) monitoring, nursing care, laboratory examinations, and antibiotics. However, there were notable differences in the audited items between China and Australia. For example, in China, medical visit costs were included in hospital ward charges, while nursing care and ECG monitoring were calculated separately. Additionally, antibiotic costs were based on the amount of the specific types of antibiotics that were used, rather than the number of days as in Australia. In this study, we audited only the total costs of antibiotics and pathology due to the diversity of types and tests.

All costs were retrospectively audited by a blinded assessor through the Hospital Information System after patient discharge. All medical interventions performed during hospitalization were decided by their blinded supervising physicians; the physiotherapist was not involved in any medical decision-making.

### 2.6 Cost-Effectiveness Analysis

The cost-effectiveness of the TIME program in preventing PPCs and improving QALYs was evaluated from a hospital perspective using the incremental cost-effectiveness ratio (ICER). The ICER was calculated by dividing the incremental cost (ΔC) — the net mean hospital cost difference per participant between the TIME and control groups — by the incremental effects (ΔE), which were the differences in PPCs rates 14 days post-surgery and QALYs 12 months post-surgery.

Next, ICER was utilized to assess the cost-effectiveness of the TIME program in the prevention of PPCs and the enhancement of QALYs from a hospital perspective. The incremental cost (ΔC) was the difference of net mean hospital costs per participant between the TIME group and the control group. Meanwhile, the incremental effect (ΔE) was defined as the difference in PPCs rates 14 days post-surgery and QALYs 12 months following surgery. The ICER was calculated by dividing incremental cost by incremental effects (ICER = ΔC/ΔE) [32].

**Fig. 2.**
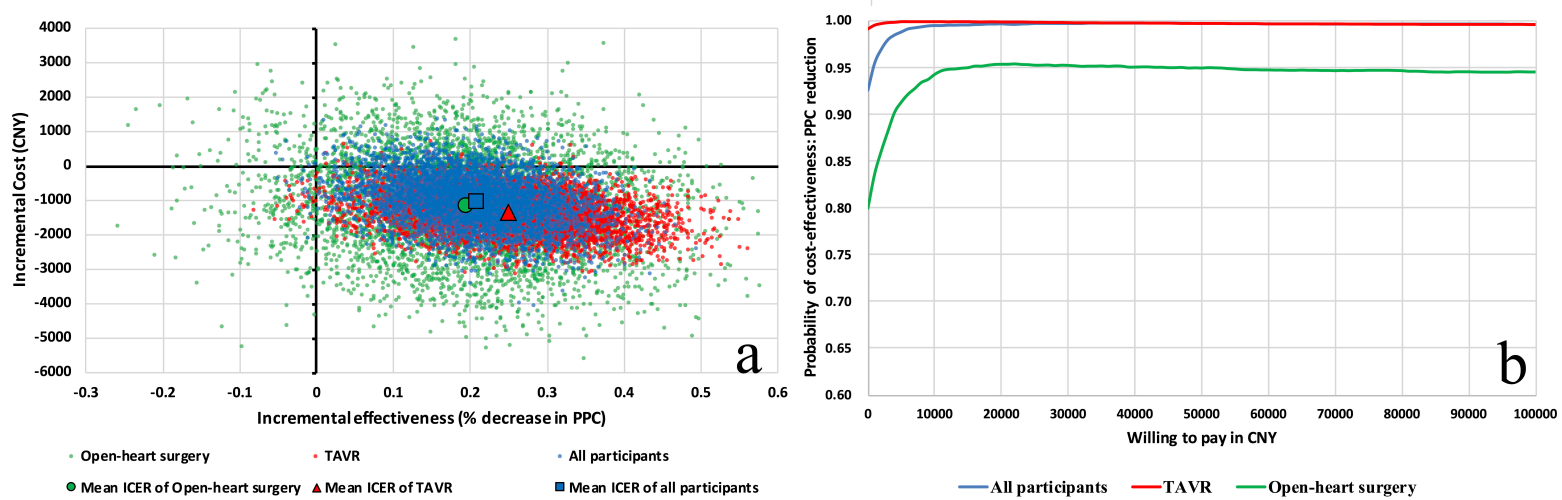
**Evaluation of cost-effectiveness for the preoperative TIME program vs. usual care in PPC prevention**. Panel (a) of Fig. 2 presents a cost-effectiveness plot comparing the preoperative TIME program with usual care in the prevention of PPCs. Points are colored to distinguish between the patient cohorts: blue for the overall patient population, red for those undergoing TAVR, and green for patients receiving open-heart surgery. The majority of blue points in the southeast quadrant indicate the TIME program’s tendency to be more effective and less costly for the overall cohort. Panel (b) shows the cost-effectiveness acceptability curve for each patient subgroup. The blue line represents the overall patient cohort with a high probability of cost-effectiveness, while the red line indicates a near-certain cost-effectiveness for TAVR patients. The green line illustrates the cost-effectiveness for open-heart surgery patients, which is lower than TAVR but still substantial. These curves represent the probability that the TIME program is cost-effective at different willingness-to-pay thresholds. TIME, three-day of inspiratory muscle training, aerobic muscle training, and specific education; PPCs, postoperative pulmonary complications; TAVR, transcatheter aortic valve replacement; ICER, incremental cost-effectiveness ratio; CNY, Chines Yuan.

Bootstrapping was used to address the large heterogeneity in the postoperative hospitalization costs. Bootstrapping referred to running the economic and clinical outcomes of the original trial through a mathematical model that uses the variance of the original data to simulate hypothetical outcomes after thousands of simulations of the trial. This approach provides a broader representation of potential cost-effective outcomes in a population, rather than relying solely on the specific trial sample. The cost-effectiveness ratios of these simulations were then plotted on a cost-effectiveness plane (Fig. 2a). The plane was divided into four quadrants, with the northeast quadrant representing more costly and more effective; the southeast quadrant representing less costly and more effective (dominance); the southwest quadrant representing less costly and less effective; and the northwest quadrant representing more costly and less effective (dominated) [33].

To address healthcare providers’ uncertainty about the value of new treatments that are more costly but improve clinical outcomes, a cost-effectiveness acceptability curve (CEAC) was utilized. The CEAC, derived from the joint density of incremental costs and effects, represents the likelihood of the intervention being cost-effective under various payment thresholds [33].

### 2.7 Statistical Analysis

IBM SPSS version 24.0 (IBM Corp., Armonk, NY, USA) was used for data analysis in the RCT. All continuous data were presented as mean (SD) and normality was assessed through the Kolmogorov-Smirnov test. The prevalence of PPCs and pneumonia were examined by the χ^2^ test. Student’s *t* test was used to examine the difference of costs between groups. All probability values were two-sided, with values <0.05 considered statistically significant. Because of heterogeneity in medical resource usage, differences in costs and effectiveness (PPCs reduction and QALYs) between groups, along with associated 95% confidence intervals, were analyzed using bootstrapping resampling. A total of 5000 paired ICER estimates were bootstrapped and presented on a cost-effectiveness plane. These estimates were further transformed into net benefits to generate the CEAC.

## 3. Results

### 3.1 Baseline Characteristics

A total of 165 patients were randomized into two groups: the preoperative TIME (intervention group, n = 82) and standard care (control group, n = 83). The detailed baseline characteristics are presented in Table 1. In the TIME group, 45 (55%) were male, with 46 (56%) receiving transcatheter aortic valve replacement (TAVR). Similarly, in the usual care group, 44 (53%) were male, with 51 (61%) undergoing TAVR.

**Table 1.** **Baseline characteristics of the study population**.

		Preoperative TIME	Usual care
		(n = 82)	(n = 83)
Age, mean (SD), y		63 (11)	63 (10)
Female, n (%)		37 (45%)	39 (47%)
Height, mean (SD), cm		161 (8)	160 (7)
Weight, mean (SD), kg		62 (11)	60 (10)
BMI, mean (SD), kg/m^2^		24.0 (3.7)	23.4 (3.3)
History of smoke			
	No smoking, n (%)	54 (66%)	44 (53%)
	Cessation of smoking, n (%)	22 (27%)	30 (36%)
	Smoking, n (%)	6 (7%)	9 (11%)
NYHA classification			
	II, n (%)	45 (55%)	29 (35%)
	III, n (%)	37 (45%)	54 (65%)
Euro Score, mean (SD)		5.2 (3.0)	5.4 (3.5)
LVEF, mean (SD), %		57.6 (11.1)	55.7 (14.7)
KCCQ, mean (SD)		62.1 (12.9)	60.0 (13.9)
Pulmonary symptoms			
	Cough, n (%)	36 (44%)	27 (33%)
	Expectoration, n (%)	19 (23%)	23 (28%)
	Wheezing, n (%)	2 (2%)	9 (11%)
	Dyspnea, n (%)	3 (4%)	8 (10%)
	Bronchial medication, n (%)	0 (0)	6 (7%)
Comorbidities			
	Hypertension, n (%)	37 (45%)	34 (41%)
	COPD, n (%)	25 (30%)	34 (41%)
	Asthma, n (%)	0 (0)	0 (0)
	Sleep apnea, n (%)	0 (0)	0 (0)
	Inspiratory muscle weakness, n (%)	53 (65%)	56 (67%)
	Coronary heart disease, n (%)	12 (15%)	19 (23%)
	Respiratory infection in the last month, n (%)	4 (5%)	7 (8%)
	Diabetes mellitus, n (%)	8 (10%)	14 (17%)
	Neurological disorders, n (%)	13 (16%)	12 (14%)
	History of median sternotomy, n (%)	5 (7%)	5(6%)
Surgical approach			
	TAVR, n (%)	46 (56)	51 (61%)
	Open-heart surgery, n (%)	36 (44%)	32 (39%)

TIME, three-day of inspiratory muscle training, aerobic muscle training, and specific education; SD, standard deviation; BMI, body mass index; NYHA, New York Heart Association; LVEF, left ventricular ejection fraction; KCCQ, Kansas City Cardiomyopathy Questionnaire; COPD, chronic obstructive pulmonary diseases; TAVR, transcatheter aortic valve replacement.

### 3.2 Outcomes of the Randomized Controlled Trial

Detailed outcomes of the randomized controlled trial are presented in Table 2. In the intervention group, the incidence of PPCs was 31.7% (26/82), significantly lower than the 53.0% (44/83) observed in control group, demonstrating statistical significance (odds ratio [OR] 0.41; 95% confidence interval [CI] 0.22–0.78). However, there were no significant differences between groups in terms of the converted EQ-5D scores (0.926 versus 0.921, 95% CI –0.019 to 0.033) 4 weeks after surgery or the estimated QALYs (0.909 versus 0.898, 95% CI –0.013 to 0.034) 12 months after surgery.

**Table 2.** **Comparative efficacy and cost-effectiveness of preoperative TIME program vs. usual care**.

		Preoperative TIME	Usual care	Odds Ratio, MD	*p*-value
		(n = 82)	(n = 83)	(95% CI)	
PPCs (n/%)					
	TAVR	8 (17.4)	22 (43.1)	0.28 (0.11 to 0.71)	0.01
	Surgery	18 (50.0)	22 (68.8)	0.46 (0.17 to 1.23)	0.12
	All participants	26 (31.71)	44 (53.01)	0.41 (0.22 to 0.78)	0.01
Pneumonia (n/%)		11 (13.4)	35 (42.2)	0.21 (0.10 to 0.46)	0.00
Mean (SD) EQ-5D					
	Baseline	0.893 (0.104)	0.885 (0.114)	0.008 (–0.028 to 0.043)	0.67
	1 month	0.926 (0.114)	0.921(0.125)	0.005 (–0.019 to 0.033)	0.80
Mean (SD) QALYs					
	TAVR	0.907 (0.09)	0.899 (0.08)	0.008 (–0.038 to 0.054)	0.97
	Surgery	0.912 (0.07)	0.898 (0.08)	0.014 (–0.040 to 0.068)	0.90
	All participants	0.909 (0.08)	0.898 (0.07)	0.011 (–0.013 to 0.034)	0.85
Mean (SD) LOS					
	ICU	1.9 (1.4)	2.4 (1.6)	–0.5 (–1.0 to 0)	0.04
	Cardiac ward	5.4 (2.3)	6.0 (3.2)	–0.6 (–1.5 to 0.3)	0.22
Costs					
	TAVR	6911 (1484)	8266 (3373)	–1355 (–3334 to 624)	0.29
	Surgery	10,494 (3683)	11,835 (6071)	–1340 (–3725 to 1044)	0.47
	All participants	8484 (3207)	9615 (4869)	–1131 (–2403 to 140)	0.08

TIME, three-day of inspiratory muscle training, aerobic muscle training, and specific education; MD, mean difference; SD, standard deviation; PPCs, postoperative pulmonary complications; TAVR, transcatheter aortic valve replacement; EQ-5D, EuroQol five-dimensional questionnaire; QALYs, quality adjusted life years; LOS, length of stay; ICU, intensive care unit.

### 3.3 Postoperative Hospital Costs

The detailed costs and use of postoperative hospital resources were presented in Table 3. The mean cost reduction in the intervention group was 1131 CNY (95% CI –2403 to 140) below the control group, with total costs for the intervention group amounting to 8484 CNY (3207) and costs for the usual care reaching 9615 CNY (4869), with no significant difference between the treatments. However, significant cost savings were observed in the intervention group for specific resources: antibiotics (339 versus 667 CNY, 95% CI –605 to –51), nursing (1021 versus 1200 CNY, 95% CI –330 to –28), and ECG monitoring (685 versus 929 CNY, 95% CI –421 to –67), while no significant difference was observed in pathology costs (3825 vs. 4449 CNY, 95% CI –1299 to 51).

**Table 3.** **Comparison of postoperative hospital resource utilisation and associated costs: preoperative TIME program vs. usual care**.

			TIME (unit)	TIME (cost)	Control (unit)	Control (cost)	Difference between groups	*p*-value
			Mean (SD)	Mean (SD)	Mean (SD)	Mean (SD)	Mean (95% CI)	
TIME program				600		0		
Hospital ward use								
	ICU stay	265/d	1.9 (1.4)	501 (368)	2.4 (1.6)	635 (416)	–134 (–262 to –6)	0.04
	Surgical stay	79/d	5.4 (2.3)	430 (184)	6 (3.2)	475 (253)	–45 (–117 to 27)	0.22
Respiratory support								
	MV	15/h	7.9 (12.2)	118 (183)	9.7 (18.7)	145 (281)	–27 (–104 to 50)	0.49
	NIV	13/h	13.5 (21.6)	175 (281)	23.8 (32.4)	309 (422)	–134 (–250 to –17)	0.03
	Oxygen	40/d	5.7 (2.4)	229 (95)	5.8 (3.5)	234 (138)	–5 (–43 to 34)	0.80
Imaging								
	X-ray	70.3/test	1.6 (0.9)	113 (62)	2 (1.3)	139 (94)	–26 (–52 to 0)	0.05
	CT	250/test	0.4 (0.7)	105 (176)	0.3 (0.5)	72 (121)	33 (–16 to 82)	0.19
	HRCT	500/test	0.6 (0.6)	291 (287)	0.6 (0.5)	315 (257)	–25 (–113 to 64)	0.59
	US	45/test	1.7 (0.9)	76 (43)	1.6 (1)	73 (46)	3 (–11 to 18)	0.66
Nursing				1021 (381)		1200 (532)	–179 (–330 to –28)	0.02
ECG monitoring		8/h	86 (57)	685 (458)	116 (77)	929 (616)	–244 (–421 to –67)	0.01
Pathology				3825 (1693)		4449 (2394)	–624 (–1299 to 51)	0.07
Antibiotics				339 (577)		667 (1056)	–328 (–605 to –51)	0.02
Total cost				8484 (3207)		9615 (4869)	–1131 (–2403 to 140)	0.08

All costs were in Chinese Yuan (CNY) for the year 2022 (the average exchange rate: 1 US dollar = 6.73 CNY). TIME, three-day of inspiratory muscle training, aerobic muscle training, and specific education; SD, standard deviation; ICU, intensive care unit; MV, mechanical ventilation; NIV, non-invasive ventilation; CT, computed tomography; HRCT, high-resolution computed tomography; US, ultrasonography; ECG monitoring, electrocardiograph monitoring.

### 3.4 Cost-Effectiveness for PPCs Reduction

In Fig. 2a, the majority of data points (blue) were positioned in the southeast quadrant, illustrating the TIME program’s dual benefits of increased effectiveness and lower costs in preventing PPCs, with a 92.6% probability of cost-effectiveness compared to usual care, as highlighted by the blue line in Fig. 2b. The distribution of data points for TAVR (red) and Open-heart surgery (green) patients shows a similar pattern. The CEAC indicated a 99% probability of cost-effectiveness in TAVR patients and 80% in Open-heart surgery patients (Fig. 2b), showcasing the substantial economic and clinical benefits of the TIME program across different surgical approaches.

### 3.5 Cost-Effectiveness for QALYs

**Fig. 3.**
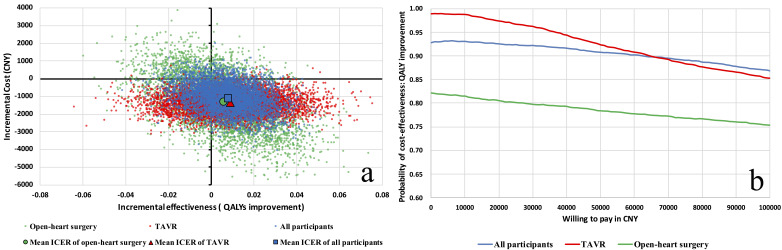
**Evaluation of cost-effectiveness for the preoperative TIME program vs. usual care for QALY enhancement**. Panel (a) offers a cost-utility plot that compares the preoperative TIME program with usual care in improving QALYs, with scatter points distributed mainly in the southeast and southwest quadrants. This distribution suggests potential cost savings despite uncertain QALY gains, with blue representing the complete patient set. Panel (b) displays the cost-effectiveness acceptability curve for the TIME program, where the blue line signals a 93% probability of cost-effectiveness for the overall group. For subgroup analysis, red denotes TAVR patients, with a 99% probability of cost-effectiveness, and green represents open-heart surgery patients, indicating an 82% probability. These findings underscore the economic and health-related benefits of the TIME program across different surgical interventions. TIME, three-day of inspiratory muscle training, aerobic muscle training, and specific education; TAVR, transcatheter aortic valve replacement; QALYs, quality adjusted life years; ICER, incremental cost-effectiveness ratio; CNY, Chinese Yuan.

In Fig. 3a, it is evident that the scatter points are largely concentrated in the southeast and southwest quadrants, reflecting the uncertainty in QALYs improvement, yet indicating a probable cost reduction associated with the TIME program. The CEAC (Fig. 3b) suggests a 93% probability of the TIME program being cost-effective for QALYs improvement. Subgroup analysis showed a 99% probability of cost-effectiveness in TAVR patients and 82% chance in patients undergoing open-heart surgery, assuming cost neutrality. Due to the uncertainty for improving QALYs, the probability of cost-effectiveness diminishes as the willingness-to-pay values increase (Fig. 3b). Overall, the hospital cost savings was the primary factor driving cost-effectiveness.

## 4. Discussion

To the best of our knowledge, this study was the first economic evaluation of a prehabilitation program for VHD patients. The results indicate that the preoperative TIME program not only reduced postoperative hospital resource usage, but also demonstrated a high probability of cost-effectiveness in preventing PPC in patients undergoing heart valve surgery. Although no statistically significant difference was found in improving QALYs, the CEAC showed a 92.8% probability that the preoperative TIME program is effective in improving QALYs without increasing costs. These benefits were primarily attributed to substantial net cost saving.

Our study’s major finding is that the significant cost savings seen in the intervention group were largely driven by a reduction in high-level care requirements, particularly nursing, ECG monitoring, antibiotic use, and pathology needs, which were cumulatively associated with a lower incidence of PPCs. PPCs not only increase hospital resource utilization but also impose significant financial burdens [34, 35]. Despite various quality improvement initiatives, such as the Surgical Care Improvement Project [6] and the ERAS protocol [21], the financial burden of PPCs persists. The importance of our findings is emphasized by previous studies which have highlighted the elevated costs associated with PPCs in cardiac surgery. Supporting this, a US national cohort study reported an average increase of $24,500 in patient-level costs due to pulmonary infections, contributing an additional $120 million to overall elective cardiac surgery costs [35]. Another study reported significantly higher hospitalization and 90-day costs associated with pneumonia in patients undergoing heart valve surgery [34]. These results suggest the preoperative TIME program has the potential to reduce hospital resource consumption by preventing PPCs in VHD patients in the Chinese healthcare system.

The impact of prehabilitation on HRQoL remains a point of contention [19, 36]. While our study did not conclusively determine the impact of the preoperative TIME program on HRQoL, the observed decrease in postoperative hospital costs provides strong evidence of its effectiveness. This supports the notion that prehabilitation, through improving preoperative functional status, can yield superior postoperative outcomes, potentially reducing long-term healthcare costs and enhancing patient quality of life [18, 37, 38]. Such findings spotlight prehabilitation’s dual potential to boost postoperative HRQoL and to streamline healthcare resource use.

The study contributes to the understanding of the growing trend towards minimally invasive interventions in VHD [39], which are linked to fewer PPCs and greater cost-effectiveness [9, 40, 41, 42, 43]. The data indicate that the preoperative TIME program could further enhance these benefits, particularly in the context of TAVR. This highlighted the importance of considering prehabilitation in the economic evaluation of TAVR as well. Interestingly, our study did not find a significant reduction in PPCs among patients undergoing open-heart surgery. This suggests that factors unique to the procedure, such as cardiopulmonary bypass and postoperative pain [10], may not benefit as much from preoperative rehabilitation. However, the associated cost savings for the hospital and demonstrated cost-effectiveness indicate advantages from the TIME program in this patient group.

Despite the growing body of evidence supporting the efficacy of prehabilitation, its clinical implementation in China remains limited, primarily due to cost and resource constraints, with expenses ranging from US$50 to US$600 [23, 24, 44], and potential increases when accounting for transportation and equipment. However, theoretical models have demonstrated that prehabilitation is cost-effective, with persistent benefits seen even at higher levels of expenditure [44, 45]. Due to the lack of community medical resources in China, extensive home-based prehabilitation is difficult to implement.

Shifting to more condensed, center-based prehabilitation programs, often limited to a 3–5-day period prior to surgery, may be necessary. Notably, previous studies have demonstrated benefits from short or even single sessions of prehabilitation [46, 47]. Our findings advocate for incorporating prehabilitation into standard preoperative care as even short-term interventions can decrease the utilization of limited resources, and result in significant cost reductions. This is especially important in resource-limited settings where prehabilitation may not be readily available. We encourage policymakers to facilitate prehabilitation’s integration into healthcare policies and reimbursement systems to increase its adoption and ensure equitable healthcare.

There are several limitations to this study. First, its participant pool was largely drawn from a single center, with the majority concentrated in China’s southwestern region. Given the regional variability in postoperative outcomes of VHD patients in China [48], our results may not be generalized to other populations. However, it is important to note that the efficacy of prehabilitation in preventing PPCs has been demonstrated across several regions [46]. Second, our study had a relatively small sample size, a common challenge associated with health economic analyses among RCTs. Although our sensitivity analysis supports the cost-effectiveness of the preoperative TIME program for both TAVR and open-heart surgery recipients, caution is warranted when interpreting these outcomes, as the results are derived from a limited sample. Third, our study’s follow-up period was constrained to only 4 weeks, and we relied on a prognostic model to estimate the one year postoperative quality of life, rather than direct data. Despite the model’s previous application to abdominal surgery patients [24], who share similar postoperative recovery patters with cardiac surgery patients [29], it’s ability to accurately reflect the postoperative health status of our cohort may be limited. Finally, the economic analysis focused exclusively on hospital expenses, omitting broader, long-term healthcare expenses. This oversight narrows our insights into other potential long-term health benefits and healthcare resource savings resulting from reduced PPCs. However, our study successfully demonstrates the short-term economic advantages of the preoperative TIME program. Future studies should extend to evaluating long-term healthcare expenditures among cardiac surgery patients, offering a more comprehensive representation of the economic and clinical benefits of prehabilitation.

## 5. Conclusions

The preoperative TIME program has been linked to lower postoperative hospital costs. Furthermore, the program demonstrated a high probability of being both cost-effective in preventing PPCs and enhancing QALYs in Chinese patients with VHD who are undergoing TAVR and open-heart surgery. These findings suggest that the TIME program could be an effective strategy for PPC prevention and cost reduction in postoperative care for this patient population.

## Availability of Data and Materials

The corresponding author can provide the datasets used and/or analyzed during the current study upon reasonable request.

## Supplementary Material

Supplementary material associated with this article can be found, in the online version, at https://doi.org/10.31083/j.rcm2509323.
