# Supplementary material for: A Three-Day Prehabilitation Program is Cost-Effective for Preventing Pulmonary Complications after Heart Valve Surgery: A Health Economic Analysis of a Randomized Trial
Source: Rev Cardiovasc Med. 2024 Sep 10;25(9):323. doi: 10.31083/j.rcm2509323 (PMC11440420; doi:10.31083/j.rcm2509323)
Supplement: Supplementary file 1 [file 2153-8174-25-9-323-s1.docx]

**Supplementary File 1**

**Table 1. Definitions of Postoperative Pulmonary Complications[1]**

| **Grade 1**   - Cough, dry - Microatelectasis: abnormal lung findings and temperature >37.5°C without other documented cause; results of chest radiograph either normal or unavailable - Dyspnea, not due to other documented cause |
| --- |
| **Grade 2**   - Cough, productive, not due to other documented cause - Bronchospasm: new wheezing or preexistent wheezing resulting in change therapy - Hypoxemia: alveolar-arterial gradient >29 and symptoms of dyspnea or wheezing - Atelectasis: radiological confirmation plus either temperature >37.5°C or abnormal lung findings - Hypercarbia, transient, requiring treatment, such as naloxone or increased manual or mechanical ventilation - Adverse reaction to pulmonary medication |
| **Grade 3**   - Pleural effusion, resulting in thoracentesis - Pneumonia, suspected: radiological evidence without bacteriological confirmation - Pneumonia, proved: radiological evidence and documentation of pathological organism by Gram stain or culture - Pneumothorax - Reintubation postoperative or intubation, period of ventilator dependence does not exceed 48 hours |
| **Grade 4**   - Ventilatory failure: postoperative ventilator dependence exceeding 48 hours, or reintubation with subsequent period of ventilator dependence exceeding 48 hours |

**References**

1. Kroenke, K., et al., *Operative risk in patients with severe obstructive pulmonary disease.* Archives of internal medicine, 1992. **152**(5): p. 967-971.
